# In Vivo Toxicity and Pharmacokinetics of Polytetrafluoroethylene Microplastics in ICR Mice

**DOI:** 10.3390/polym14112220

**Published:** 2022-05-30

**Authors:** Sijoon Lee, Kyung-Ku Kang, Soo-Eun Sung, Joo-Hee Choi, Minkyoung Sung, Keum-Yong Seong, Jian Lee, Subin Kang, Seong Yun Yang, Sunjong Lee, Kyeong-Ryoon Lee, Min-Soo Seo, KilSoo Kim

**Affiliations:** 1 Preclinical Research Center, Daegu-Gyeongbuk Medical Innovation Foundation, Daegu 41061, Korea; sjlee1013@kmedihub.re.kr (S.L.); kangkk@kmedihub.re.kr (K.-K.K.); sesung@kmedihub.re.kr (S.-E.S.); cjh522@kmedihub.re.kr (J.-H.C.); tjdalsrud27@naver.com (M.S.); 2 Institute of Animal Medicine & Department of Veterinary Medicine, Gyeongsang National University, Jinju 52828, Korea; 3 Department of Biomaterials Science (BK21 Four Program), Life and Industry Convergence Institute, Pusan National University, Miryang 50463, Korea; ky.seong0124@gmail.com (K.-Y.S.); paperin163@gmail.com (J.L.); kangsb1789@gmail.com (S.K.); syang@pusan.ac.kr (S.Y.Y.); 4 Korea Institute of Industrial Technology, Cheonan 31056, Korea; sunjong1774@kitech.re.kr; 5 Laboratory Animal Resource Center, Korea Research Institute of Bioscience and Biotechnology, Cheongju 28116, Korea; kyeongrlee@kribb.re.kr; 6 Department of Bioscience, University of Science and Technology, Daejeon 34113, Korea; 7 College of Veterinary Medicine, Kyungpook National University, 80 Dahakro, Buk-gu, Daegu 41566, Korea

**Keywords:** microplastics, polytetrafluoroethylene, toxicity evaluation, pharmacokinetics

## Abstract

The increased use of plastics has led to severe environmental pollution, particularly by microplastics—plastic particles 5 mm or less in diameter. These particles are formed by environmental factors such as weathering and ultraviolet irradiation, thereby making environmental pollution worse. This environmental pollution intensifies human exposure to microplastics via food chains. Despite potential negative effects, few toxicity assessments on microplastics are available. In this study, two sizes of polytetrafluoroethylene (PTFE) microplastics, approximately 5 μm and 10–50 μm, were manufactured and used for single and four-week repeated toxicity and pharmacokinetic studies. Toxicological effects were comprehensively evaluated with clinical signs, body weight, food and water consumption, necropsy findings, and histopathological and clinical-pathological examinations. Blood collected at 15, 30 60, and 120 min after a single administration of microplastics were analyzed by Raman spectroscopy. In the toxicity evaluation of single and four-week repeated oral administration of PTFE microplastics, no toxic changes were observed. Therefore, the lethal dose 50 (LD_50_) and no-observed-adverse-effect-level (NOAEL) of PTFE microplastics in ICR mice were established as 2000 mg/kg or more. PTFE microplastics were not detected in blood, so pharmacokinetic parameters could not be calculated. This study provides new insight into the long-term toxicity and pharmacokinetics of PTFE microplastics.

## 1. Introduction

Globally, the amount of plastic used is steadily increasing, with production rising from 1.5 million tons in the 1950s to 3.35 billion tons in 2016 [1]. Some plastics are recycled, but most are discarded, often causing environmental pollution. Plastic particles with a size of less than 5 mm are called microplastics [2,3]. These small particles are designated as one of the world’s four major environmental issues, along with environmental changes, ozone layer destruction, and marine oxidation [4]. Microplastics are classified as primary microplastics made for use in personal care products, cosmetics, toothpaste, detergents, sunscreens, and drug vectors [5,6,7]. Secondary microplastics are formed by environmental factors, such as UV oxidation, light degradation, and physical ablation [5,8,9,10]. Primary microplastics are mainly spherical in keeping with their uses, and secondary microplastics are mostly fractured or fibrous forms due to weathering and other environmental factors. The latter particles cause environmental pollution worldwide [11,12]. Studies on rivers and seas detect large amounts of microplastics [13,14,15]. In addition, secondary microplastics are small, making them difficult to remove during sewage treatment [16]. Thus, particles are released into receiving waters and eventually flow into the seas. These microplastics may stick to the gills of aquatic organisms during oxygen exchange or when particles are mistaken for food [17]. Subsequently, microplastics accumulate in these aquatic organisms and can be detected in various ways [18,19]. The consumption of aquatic organisms that have accumulated microplastics might be harmful to humans, making toxicological research essential. Further, many studies report microplastics of various sizes, colors, and types, such as polyethylene (PE), polypropylene (PP), and polyrthylene terephthalate (PET), in easily accessible foods (mineral water, salt, beer, wine, canned foods, milk, seaweed) [20,21,22,23,24,25,26,27]. Exposure to microplastics can occur via inhalation, dermal absorption, or ingestion, with the ingestion route being the most important [28]. In vitro, microplastics show increased accumulation and toxicity through the inhibition of ATP-binding cassette transporter activity in Caco-2 cells [29] and significantly increase the production of ROS in human-derived HDFs, PBMCs, and Raw 264.7 cells [30]. Oxidative stress increased in human cerebral cells (T98G) and epitaxial cells (HeLa) after exposure to microplastics [31]. These impacts at the cellular level suggest that microplastics might exhibit toxicity in humans. Experiments at this cell level suggest that microplastics can potentially exhibit influence or toxicity in humans. Recently, in vivo experiments using microplastics are on the rise. In aquatic organisms, PE and PP caused increased mortality and gut clearance times and decreased growth of Hyalella azteca [32]. Polystyrene (PS) caused reduced body weight, body length, and body mass index and increased inflammatory cytokine and chemokine gene expression in zebrafish [33]. In oysters, a decrease in the number and diameter of female oocytes, the speed and activity of male sperm, and the growth rate of larval were observed [34]. In experiments using mammals, PE also altered intestinal microbial populations in C57BL/6 mice and cause inflammatory cell infiltration and loosening of glands in the colon [35]. PS caused inflammation of testicles, fibrosis of ovaries, adipose metabolic disorders in the liver, a reduction of mucin secretion in the colon, and a change in the intestinal microbial population in intestinal microorganisms [36,37,38,39]. PS is distributed to the liver, kidney, and gastrointestinal tract, and affects energy metabolism, lipid metabolism, oxidative stress, and neurological function [40]. Additionally, our previous study showed that polyethylene microplastics can induce granulomatous inflammation in the lungs after four-week repeated oral administrations [41]. Thus, microplastics may be toxic to various tissues, including reproductive and nervous systems. PTFE is a plastic with a density of 2.0 g/m^3^ and 2.3 g/m^3^ for amorphous and crystalline portions, respectively [42]. Unlike general plastics, it is used to coat kitchen utensils applied via spraying. Most of these kitchenware coatings can peel off during cooking through heating or friction with utensils [43]. Released PTFE can be incorporated into cooked food and subsequently ingested. Despite this high potential for human exposure, the number of animal experiments using microplastics is still small, and most animal experiments used microplastics composed of polystyrene and polyethylene. PTFE is rarely studied in vivo for toxicity and kinetics in mammals. We manufactured two sizes of PTFE microplastics to confirm whether the manufactured microplastics conform to the commonly used definition of microplastics, under the size of 5 mm. A typical toxicity test was conducted using manufactured microplastics to establish LD_50_ and NOAEL, and a pharmacokinetic experiment was conducted to determine the organs in which microplastics are widely distributed in the body.

## 2. Materials and Methods

### 2.1. Preparation of Polytetrafluoroethylene Microplastics

Two sizes of PTFE microplastics (approximately 5 μm and 10–50 μm) were made from PTFE raw materials (TF1641, Dyneon^TM^, Aston, PA, USA). For the preparation of PTFE 10~50 μm particles, PTFE was frozen at −78 °C with dry ice and ground using a blade-type homogenizer for 4–5 h. Particles were filtered sequentially through 63 μm and 10 μm mesh filters and washed 4–5 times with ethanol. Particles were then dried in an oven at 50 °C for 48 h. For the preparation of approximately 5 μm PTFE particles, 10~50 μm particles of PTFE were dispersed in ethanol and ground with a high-pressure homogenizer 4 times with 600 bar. Then we separated particles sequentially using a 15 μm mesh filter and a 5 μm mesh filter and washed particles with ethanol 4~5 times. Finally, the particles were dried for 48 h in a 50 °C oven.

### 2.2. Characterization of Polytetrafluoroethylene Microplastics

The average particle size was measured using a Particle Size Analyzer based on light scattering (PSA, ELS-Z2Plus, Otsuka Electronics, Osaka, Japan). This method can be used to obtain the hydrodynamic size of a particle using the Stokes–Einstein relationship by analyzing the fluctuation of scattered light by suspended particles when illuminated with a laser to determine the rate of Brownian motion. The shape of microplastics was confirmed using a 3D-laser profile (Confocal microscopy, Keyence, Itasca, IL, USA) and scanning electron microscopy (SEM, JSM-6701F, JEOL Inc., Akishima, Tokyo, Japan). Finally, chemical identity was confirmed using a Raman microscope (RAMANtouch, Nanophoton, Osaka, Japan) equipped with a 532 nm-laser diode. The morphology of microplastics was examined with a 20× objective lens (Nikon LU Plan Flour 20×/0.45), then Raman spectra were collected in the 45.3–4273.8 cm^−1^ range using 300 lines per mm grating with a 50 µm slit width. Spectra were measured over a 16-bit dynamic range with Peltier cooled charge-coupled device detectors. The laser power was adjusted to 2 mW for each scan to obtain a sufficient signal. The acquisition time and the number of accumulations were adjusted for each measurement to obtain sufficient data for library searching. The spectrometer was calibrated with silicon at a line of 520 cm^−1^ before spectral gaining. Raman spectra were analyzed using the SLOPP Library of Microplastics and the spectral library in KnowItAll software (Bio-Rad Laboratories, Inc., Hercules, CA, USA). Similarities above the Hit Quality Index of 80 were considered satisfactory.

### 2.3. Animal and Ethics Statement

Five-week-old specific-pathogen-free ICR mice, 128 male and 104 female, were purchased from KOATECH Inc., Pyeongtaek, Gyunggi-do, Korea. Mice were housed in a standard SPF facility in ventilated IVC cages (395W × 346D × 213H) at 22 ± 1 °C, relative humidity of 50 ± 10%, a ventilation time of 10–15 h, light for 12 h per day, and illumination of 150–300 lux. All animal care and experimental procedures were approved by the Institutional Animal Care and Use Committee of the Laboratory Animal Center of the DGMIF (IACUC; approval No. DGMIF-21031601-00) and were in accordance with their guidelines.

### 2.4. Single Oral Dose Toxicity Study

Two groups of 12 male and 12 female mice were acclimatized for 1 week to evaluate the different sizes of PTFE microplastics. Each group of animals was divided into four subgroups (control, low-dose (500 mg/kg), mid-dose (1000 mg/kg), and high-dose (2000 mg/kg)) with three male and three female animals for each subgroup. A single dose of corn oil was administered to control mice, and microplastics suspended in corn oil were administered for each of three microplastic doses. The volume of corn oil was constant at 10 mL/kg. The study was conducted with reference to OECD guideline 423. The observation of clinical signs, the presence of moribund or dead animals, and the measurement of body weight were performed once a day, twice a day, and once a week, respectively, during the two-week observation period. All animals were anesthetized with CO_2_ at the end of the study and exsanguinated through the abdominal aorta. Complete gross postmortem examination was performed on all mice.

### 2.5. Four-Week Repeated Oral Dose Toxicity Study

Two groups of 40 male and 40 female mice were acclimatized for 1 week to evaluate different sizes of PTFE microplastics. Each group of animals was divided into four subgroups (control, low-dose (500 mg/kg), mid-dose (1000 mg/kg), and high-dose (2000 mg/kg)) with ten male and ten female animals for each subgroup. Four-week repeated administration of corn oil for the control group and microplastics suspended in corn oil for each of three microplastic doses were performed. Animals in the remaining groups received microplastics suspended in corn oil to achieve the above doses for each particle size. The volume of corn oil was constant at 10 mL/kg. This study was conducted with reference to OECD guideline 408. The observation of clinical signs and the presence of moribund or dead animals were recorded once a day and twice a day, respectively. The measurement of body weight, food consumption, and water consumption were all measured once a week. After four weeks, blood was collected from the abdominal aorta under isoflurane (Hana Pharm, Co., Ltd., Seoul, Korea) anesthesia. Blood samples collected from animals were analyzed using a blood cell analyzer (ADVIA 2120i, SIEMENS, Muenchen, Germany) and a serum biochemistry analyzer (TBA 120-FR, Toshiba, Tokyo, Japan). Complete gross postmortem examinations were performed on all animals and tissues. The adrenal glands, brain, cecum, colon, duodenum, epididymis, esophagus, heart, ileum, jejunum, kidney, liver, lungs, ovary, pancreas, parathyroid glands, pituitary gland, rectum, spinal cord, spleen, stomach, testis, thymus, thyroid gland, trachea, and uterus were harvested. Organ weights were recorded for the brain, spleen, heart, kidney, liver, testis, epididymis, and ovary. Tissues were fixed in 10% neutral buffered formalin (BBC Biochemicals, Mount Vernon, WA, USA), except for testes, which were fixed in Davison’s fixative followed by storage in 10% neutral buffered formalin. A tissue processor (Thermo Fisher Scienctific, Inc., Runcorn, UK) was used to prepare fixed tissues. Four-micrometer sections were cut from paraffin-embedded tissue blocks and mounted onto glass slides. Sections were then stained with hematoxylin and eosin using an autostainer (Dako Coverstainer; Agilent, Santa Clara, CA, USA). Personnel that performed histopathological evaluations were blind to the sources of tissues.

### 2.6. Pharmacokinetics Study

Pharmacokinetics of PTFE was characterized in 24 male mice acclimatized for 1 week. Animals were divided into 2 groups with social housing of three mice per cage. Animals were administered a single oral dose of 2000 mg/kg PTFE microplastics, followed by blood collection after 15, 30, 60, and 120 min. Groups of animals were administered one of the two sizes of PTFE particles.

### 2.7. Quantitative Evaluation of Polytetrafluorethylene Microplastics in Blood

Whole blood collected at predetermined times was pooled, and a 10 wt% aqueous KOH solution (20 times sample weight) was added. These samples were incubated at 37 °C for 48 h with shaking at 250 rpm. Sample solutions were filtered stepwise through stainless-steel (47 mm disk, 45 µm pore size) and silicon filters (1 cm × 1 cm, 1 um pore size) provided by Nanophoton. The Raman microscope used RAMANtouch to assess the number of PTFE microplastics collected on silicon filters.

### 2.8. Data Analysis

A non-compartmental analysis was performed to calculate pharmacokinetic parameters for PTFE using Phoenix Winnonlin 8.1 software. The area under the plasma concentration–time curve (AUC_last_) from time zero to infinity (AUC_∞_) was calculated with linear trapezoidal and standard area extrapolation methods. Terminal half-life (t_1/2_) was calculated as 0.693/λ, where λ represents the terminal slope of the log-linear of larotrectinib concentration–time profile. Total clearance (CL) for PTFE was calculated as dose/AUC_∞_ and steady-state volume of distribution (V_ss_) was then calculated as MRT × CL. The observed maximum concentration (C_max_) and time to reach C_max_ (T_max_) were obtained directly from individual PTFE plasma concentration–time profiles.

### 2.9. Human NOAEL Dose

The human NOAEL dose was estimated using the HED (human equivalent dose) converting table for mice. A 2000 mg/kg dose, the assumed NOAEL, was used for the calculation (Equation (1)) because PTFE plasma concentrations were not recorded within the range of 500~2000 mg/kg.

Dose_human_ (mg/kg) = Dose_mouse_ (mg/kg) × (Km_mouse_/Km_human_) (1)

### 2.10. Statistical Analysis

All hematology, serum biochemistry, and body weight data are presented as means ± standard deviation. The statistical significance of the differences between treated and control animals was evaluated using Student’s *t*-tests and one-way analysis of variance with the SAS program (version 9.4, SAS Institute Inc., Cary, NC, USA).

## 3. Results

### 3.1. Characterization of Polytetrafluoroethylene Microplastics

**Figure 1:**
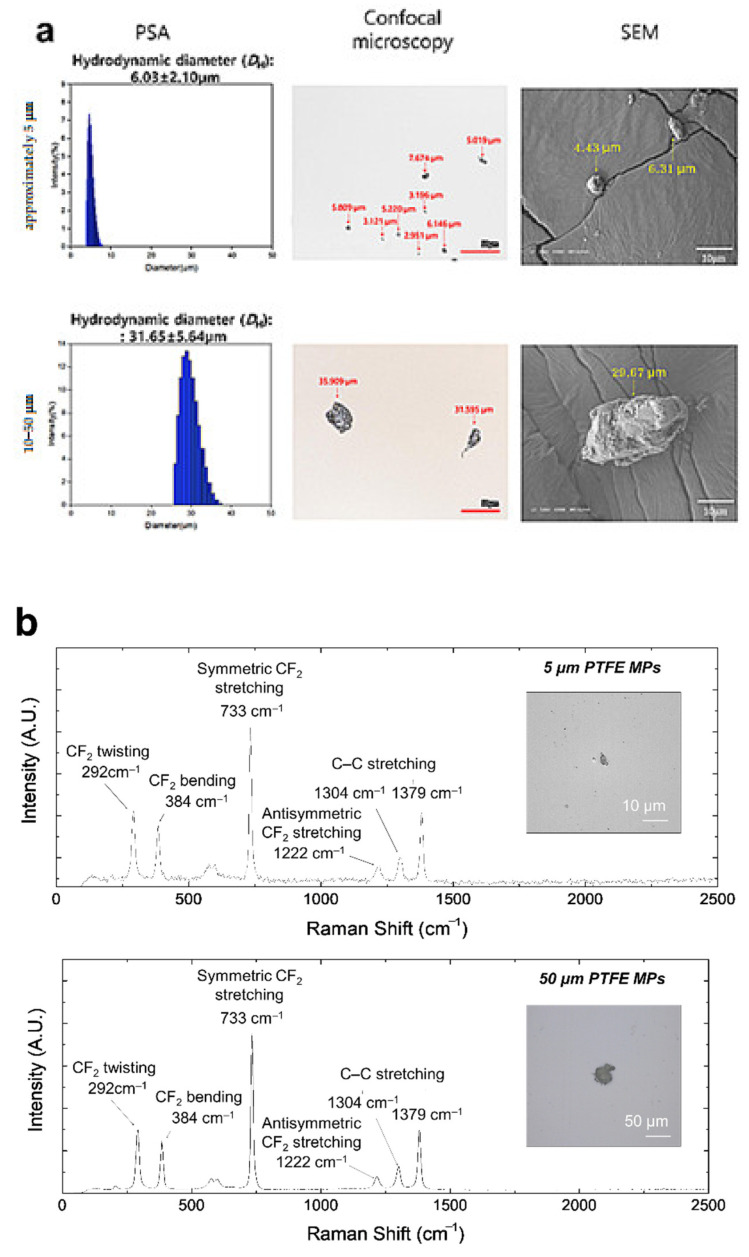
Characterization of PTFE microplastics. (**a**) Analysis with PSA, confocal microscopy, and scanning electron microscopy, (**b**) Raman spectra of approximately 5 μm and 10–50 μm-sized PTFE microplastics captured on silicon filters. Inset images show representative PTFE microplastics. Scale bar = 10 μm and 50 μm.

PSA, Confocal microscopy, SEM, and Raman microscope analysis were performed to characterize prepared PTFE microplastics and assess the intended size. PSA confirmed the two sizes of microplastics were approximately 5 μm (6.03 ± 2.10 μm) and 10–50 μm (31.65 ± 5.64 μm) (Figure 1a). The actual size of microplastics was measured with confocal microscopy and SEM (Figure 1a). Representative Raman spectra obtained from filtered microparticles identified PTFE based on peaks at 290 cm^−1^ to 1400 cm^−1^, presenting symmetric stretch CF_2_ and C–C peaks at 733 cm^−1^ and 1379 cm^−1^, and CF_2_ twisting and CF_2_ bending peaks at 292 cm^−1^ and 384 cm^−1^, respectively [44]. In addition, lines of medium intensity at 1222 cm^−1^ and 1304 cm^−1^ were ascribed to the antisymmetric stretch peak of CF_2_ [45] (Figure 1b). The physical and chemical properties of the particles prepared through these analytical methods were confirmed to be suitable for the conditions of PTFE microplastics.

### 3.2. Toxicity Study

**Figure 2:**
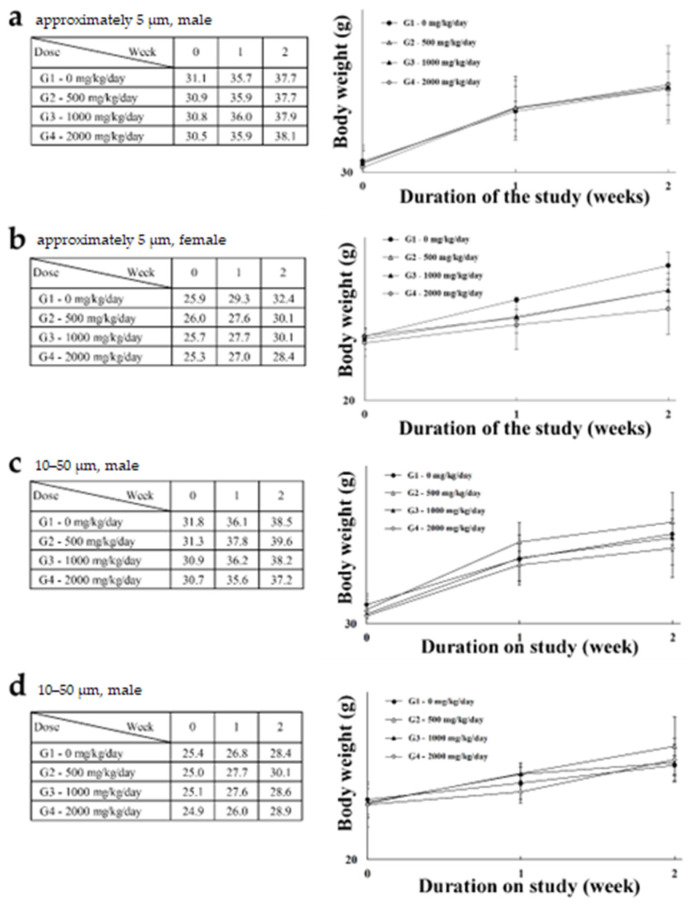
Male and female bodyweight changes after a single dose of approximately 5 μm (**a**,**b**) and 10–50 μm (**c**,**d**) PTFE microplastics.

A single-dose toxicity study was performed to identify an approximate lethal dose of PTFE microplastics in two size ranges. No morbidity or death in mice was observed, and no specific clinical symptoms were recorded. Further, no significant weight changes were associated with exposure to microplastics when compared to control animals (Figure 2a–d). At necropsy, macroscopic examinations did not reveal changes associated with PTFE microplastic administration (data not shown). Thus, the approximate lethal dose for both sizes of PTFE microplastics was 2000 mg/kg or greater. The above LD_50_ and NOAEL values suggest that the prepared PTFE microplastics show no harm to mice in vivo at a dose of less than 2000 mg/kg.

**Figure 3:**
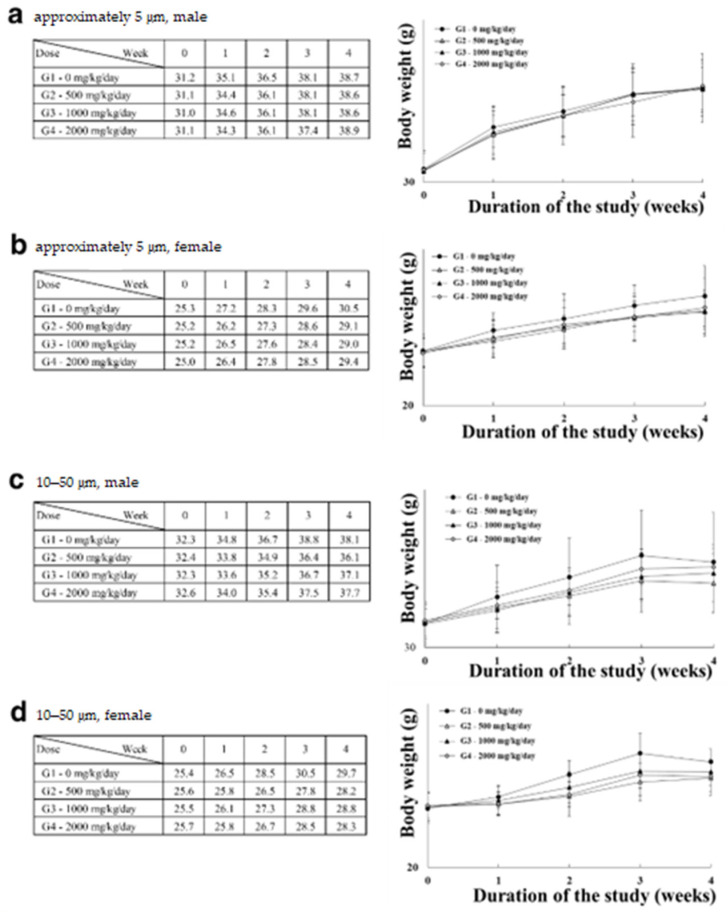
Male and female bodyweight changes after four weeks of repeated dosing with approximately 5 μm (**a**,**b**) and 10–50 μm (**c**,**d**) PTFE microplastics.

**Figure 4:**
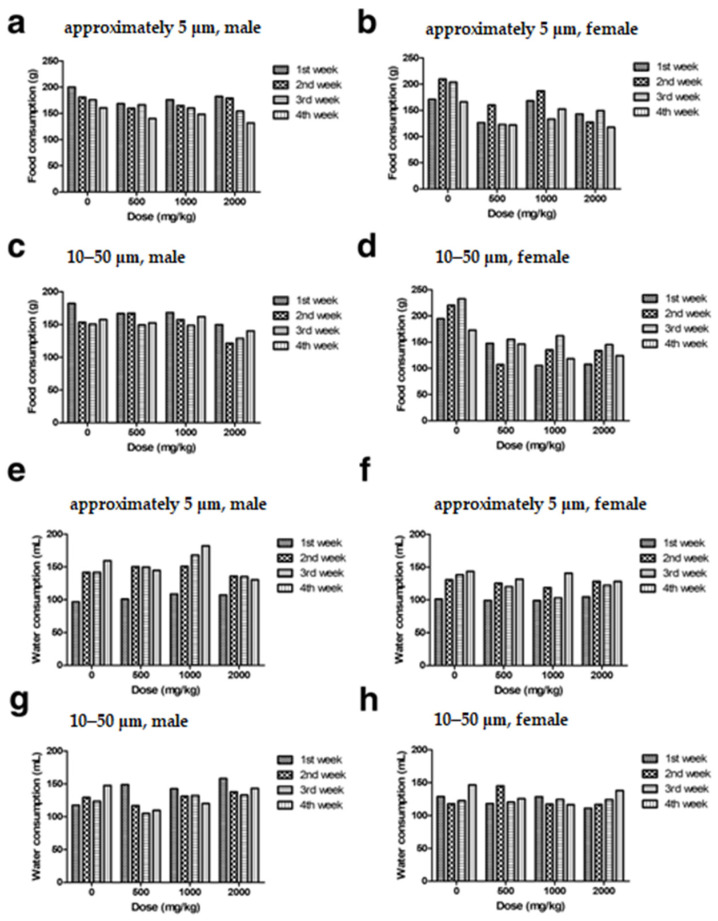
Male and female food and water consumption changes during the four-week repeated-dose toxicity study of approximately 5 μm (**a**,**b**,**e**,**f**) and 10–50 μm (**c**,**d**,**g**,**h**) PTFE microplastics.

**Figure 5:**
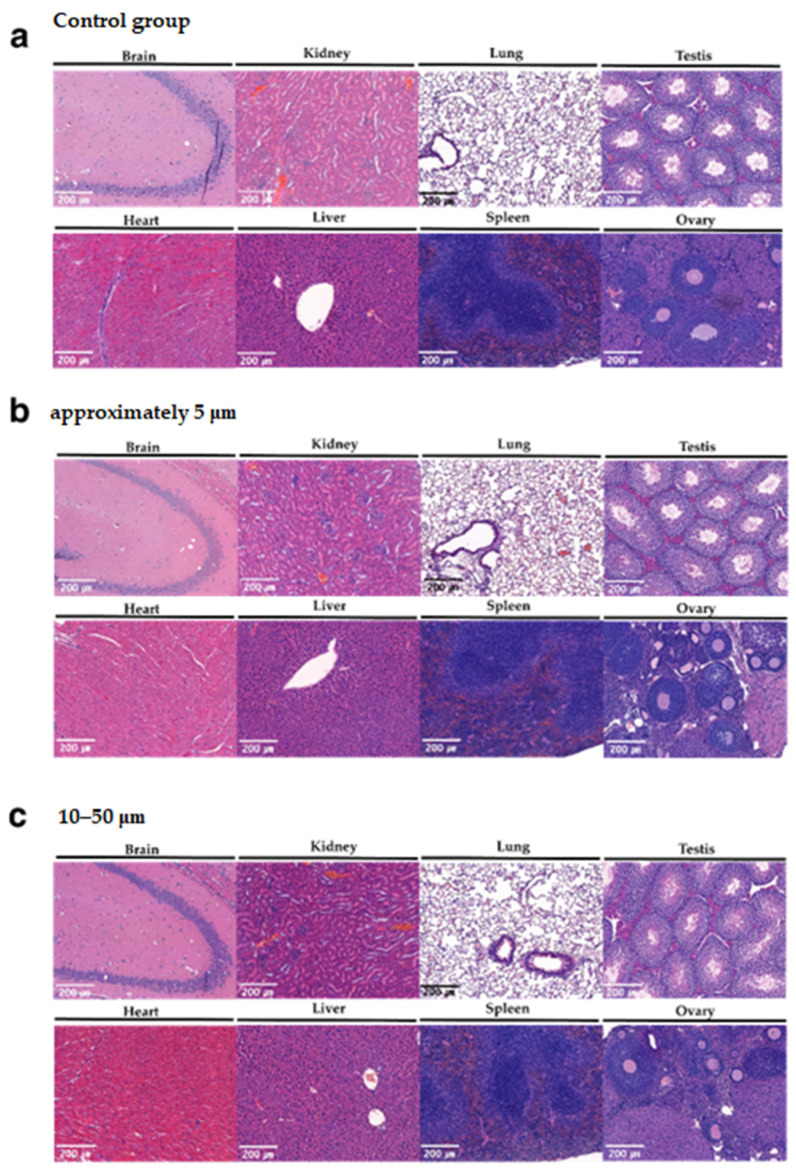
Histopathological evaluation of tissues from mice after four weeks of repeated dosing. Representative images of the brain (hippocampus), heart, kidney, liver, lung, spleen, testis, and ovary of the control mice (**a**), approximately 5 μm PTFE-treated mice (**b**) and 10–50 μm PTFE-treated mice (**c**). Scale bar = 200 μm.

Four-week repeated-dose toxicity study based on the single-dose toxicity study and OECD guideline No. 408 was then used to establish a NOAEL for chronic exposure. No morbidity or death of animals was observed, and no specific clinical signs were recorded. Body and organ weights (Figure 3a–d, Supplementary Table S1), food (Figure 4a–d) and water (Figure 4e–h) consumption, clinical pathology (Table 1), and histopathological changes (Figure 5a–c) due to PTFE administration were not observed. Thus, NOAELs for two sizes of PTFE microplastics were 2000 mg/kg or greater.

### 3.3. Pharmacokinetics Study

**Figure 6:**
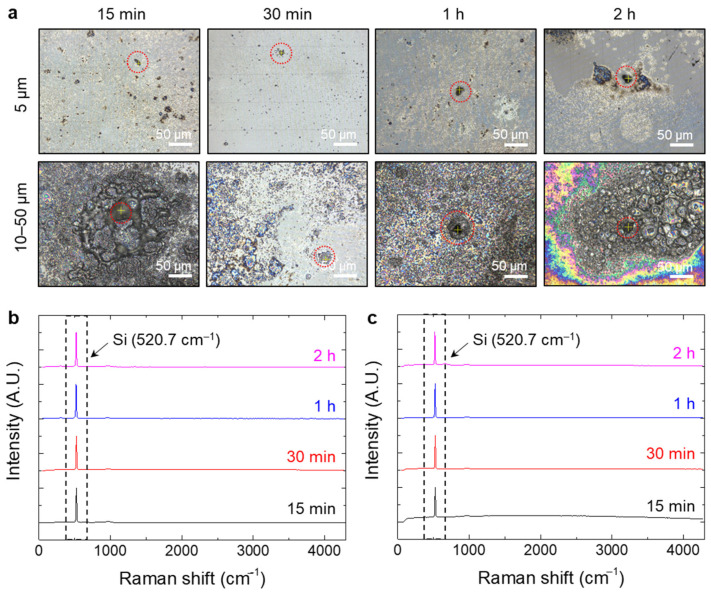
Detection of PTFE microplastics in whole blood. (**a**) Microscopic images of microparticles captured on silicon filters from blood collected at 15, 30, 60, and 120 min after oral administration of PTFE microplastics (approximately 5 and 10–50 μm). (**b**,**c**) PTFE microplastics were not detected on filters. Silicon (Si) peaks are indicated by the arrow. Scale bar = 50 μm.

Pharmacokinetic experiments were performed to determine how PTFE microplastics were distributed in blood and organs after oral administration. The distribution of PTFE microplastics was analyzed at four time points (15, 30, 60, and 120 min) after a single oral dose of microplastics. However, PTFE plasma concentrations were not detected with Raman spectrometry (Figure 6a–c), and pharmacokinetic parameters for PTFE could not be calculated.

### 3.4. Human NOAEL Dose

The human NOAEL dose could be estimated using the HED converting table and Equation (1). Assuming the NOAEL in mice is 2000 mg/kg (i.e., the highest dose in mice pharmacokinetic studies), the human NOAEL was calculated to be 9720 mg per 60 kg.

## 4. Discussion

Plastic use continues to increase worldwide. Physical properties, diversity, and low prices make the use of plastics attractive [46]. One report indicated that approximately 3% of plastic was recycled worldwide in 2017 and 67.8 million metric tons will be discarded in the environment by 2050, reflecting an increase in plastic use [47]. Discarded plastics are degraded to a size of less than 5 mm, called microplastics, via weathering and ultraviolet rays [48]. Their small size allows microplastics to move easily to contaminate soil, lakes, rivers, and seas [49,50,51,52,53,54]. In particular, many reports indicate that marine life accumulates microplastics on gills during oxygen exchange or plastics are ingested when mistaken for food [17]. Human exposure may occur when microplastic-loaded aquatic organisms are consumed [55]. Another means of human exposure is encountered when eating cooked food [56]. Microplastics are reported in tap water, beer, salt, and mineral water, and large amounts are detected in meat or cooked food in Thai markets [57,58,59]. Thus, the ingestion of contaminated food will cause human exposure to microplastics found in various aquatic organisms.

Many studies indirectly confirm such exposure to microplastics. Microplastics are detected in marine environments and aquatic organisms [60,61,62]. In vivo, aquatic organisms are often used as experimental models, and microplastics accumulate in zebrafish in the intestines, histopathological changes occur in vacuolization, and the existence of mast cells, the release of mobile cells, and other changes such as an increase in oxidative stress occur [63]. In addition, decreased movement, neurological changes such as seizures, and female reproductive dysfunction are also reported [62]. In experiments using minnows, changes such as decreased movement and increased oxidative stress and immune response are reported [64]. Female and male reproductive dysfunction appeared in oysters [34]. Experiments with mammals, especially rodents, report changes in the reproductive systems of female and male mice, behavioral changes, and liver inflammation [36,37,38]. Information is thus increasing on the risks associated with microplastics, but many knowledge gaps remain. In particular, toxicity studies have distinct limitations, such as observing only specific physiological systems or very low concentrations. Our work included a comprehensive evaluation of toxicity across all organs for PTFE particles, as recommended by OECD guidelines 408 and 423. In addition, a pharmacokinetic evaluation using Raman spectroscopy was completed to confirm in vivo behavior of PTFE.

Microplastics used in most experiments are spherical. Microplastics in the environment are more fragmented or fibrous [65]. We manufactured two sizes of fragmented microplastics, approximately 5 μm and 10–50 μm. These morphological characteristics were confirmed through PSA, confocal microscopy, and SEM (Figure 1a), and the chemical properties of PTFE were confirmed through Raman spectroscopy (Figure 1b). These analytical results suggest that manufactured particles are suitable for the physical and chemical conditions of the PTFE microplastics. Subsequently, single and four-week repeated-dose toxicity evaluations were performed using these microplastics. The single-dose toxicity test confirmed the LD_50_ of PTFE using doses of 500, 1000, and 2000 mg/kg. During the two-week observation period after a single administration, no deaths or clinical signs of toxicity were recorded (data not shown) and no weight changes (Figure 2a–d) due to PTFE microplastic administration were observed. Further, no changes due to microplastic administration were observed in necropsy. Thus, the LD_50_ for PTFE microplastics was 2000 mg/kg or more for both particle sizes.

Subsequently, no specific clinical sign was recorded during the 4-week observation period, and no significant differences were observed in body weight (Figure 3a–d), organ weight (Supplementary Table S1), or water and food consumption (Figure 4a–h) in the administration groups compared to the control group. As a result of the clinical pathological evaluation (a–h in Table 1), no dose-dependent and simultaneously significant difference was observed, and no degenerative changes such as inflammation, desquamation, or necrosis were observed in the histopathological evaluation (Figure 5a–c). Therefore, NOAEL for four-week repeated oral administration of PTFE microplastics was confirmed to be 2000 mg/kg or higher. The results provide a new understanding of acute and chronic oral exposure to PTEF microplastics.

In addition, Raman spectroscopy analysis of blood after a single dose of 2000 mg/kg showed no PTFE microplastics. The absorption of microplastics through oral administration is known to be very low [66]. In the case of weathered microplastics, if analyzed by Raman spectroscopy, the peak of microplastics may change, resulting in inaccurate results [67]. Additional experiments such as administering microplastics through IV or experiments to visually determine the bio-distribution of microplastics by labeling fluorescent substances on microplastics are necessary to overcome this limitation and directly confirm the kinetics of microplastics in vivo.

Our present study is the first to report single and repeated toxicity, along with pharmacokinetics using high concentrations of PTFE microplastics in mammals. These experiments provide new insight into the biological effects of microplastics. The difference between our previous study on the toxicity of polyethylene microplastics [41] and the results of the present study suggests that there may be differences in biological effects depending on the type of plastic. This hypothesis can be tested with in vivo experiments using other plastics, such as PS, PET, and polyamides.

## 5. Conclusions

The purpose of this study was to establish LD_50_ and NOAEL values, which are representative values of the toxicity study for two sizes of PTFE microplastics, and confirm the movement of microplastics in the body. In the single and four-week repeated toxicity study, no significant differences were observed in clinical sign, body weight, organ weight, water and food consumptions, macroscopic examination at necropsy, or clinical and histopathological evaluation. Both single and four-week repeated oral dose toxicity tests suggested LD_50_ and NOAEL values of more than 2000 mg/kg. A pharmacokinetic experiment using Raman spectroscopy analysis did not detect PTFE in blood, and pharmacokinetic parameters could not be calculated. To overcome the limitations of this experiment, pharmacokinetic evaluation using a different route of administration is required.

## Figures and Tables

**Table 1:** ` WQS. Male and female serum biochemistry hematology data of approximately 5 μm (a–d) and 10–50 μm (e–h). The results are expressed as the mean ± SD. * *p* < 0.05, ** *p* < 0.01, *** *p* < 0.001 vs. 0.

**a. Approximately 5 μm, Male, Serum Biochemistry Parameters**								
**Group/** **Dose (mg/kg/Day)**	**Sodium (mmol/L)**	**Potassium (mmol/L)**	**Chloride (mmol/L)**	**Total Protein (g/dL)**	**Albumin (g/dL)**	**Blood Urea Nitrogen** **(mg/dL)**	**Creatinine (mg/dL)**	**Glucose (mg/dL)**
G1 0	150.3 ± 1.9	7.6 ± 2.0	113.0 ± 2.7	5.0 ± 0.3	3.1 ± 0.3	22.4 ± 4.4	0.2 ± 0.0	93.0 ± 28.9
G2 500	149.7 ± 1.7	7.7 ± 1.8	114.8 ± 3.3	5.1 ± 0.1	3.3 ± 0.1	19.2 ± 4.7	0.2 ± 0.0	90.1 ± 22.1
G3 1000	151.7 ± 0.7	7.3 ± 1.1	114.7 ± 1.1	5.2 ± 0.2	3.2 ± 0.2	22.6 ± 4.9	0.2 ± 0.0	78.6 ± 15.6
G4 2000	150.7 ± 2.7	6.9 ± 1.0	113.9 ± 4.3	5.3 ± 0.2 *	3.3 ± 0.1	23.1 ± 6.7	0.2 ± 0.0	93.5 ± 51.1
**Group/** **Dose (mg/kg/Day)**	**Total Bilirubin (mg/dL)**	**Calcium (mg/dL)**	**Phosphate (mg/dL)**	**Total Cholesterol (mg/dL)**	**Triglyceride (mg/dL)**	**Aspartate Aminotransferase (U/L)**	**Alanine Aminotransferase (U/L)**	**Alkaline Phosphatase (U/L)**
G1 0	0.1 ± 0.0	8.9 ± 0.4	7.9 ± 1.1	137.2 ± 23.0	167.9 ± 53.6	69.4 ± 28.2	23.9 ± 6.5	248.6 ± 92.5
G2 500	0.1 ± 0.0	8.9 ± 0.3	7.1 ± 1.3	139.9 ± 20.6	136.6 ± 40.1	71.8 ± 35.6	27.1 ± 8.6	222.6 ± 90.3
G3 1000	0.1 ± 0.0	9.0 ± 0.2	7.8 ± 0.9	152.7 ± 23.7	140.5 ± 45.5	64.8 ± 9.8	22.9 ± 3.2	191.6 ± 65.8
G4 2000	0.1 ± 0.1	9.2 ± 0.5	7.5 ± 0.8	150.8 ± 26.1	140.7 ± 47.6	81.9 ± 53.9	30.4 ± 11.0	223.0 ± 72.4
**b. Approximately 5 μm, Female, Serum Biochemistry Parameters**								
**Group/** **Dose (mg/kg/Day)**	**Sodium (mmol/L)**	**Potassium (mmol/L)**	**Chloride (mmol/L)**	**Total Protein (g/dL)**	**Albumin (g/dL)**	**Blood Urea Nitrogen** **(mg/dL)**	**Creatinine (mg/dL)**	**Glucose (mg/dL)**
G1 0	148.7 ± 1.4	6.4 ± 1.2	112.7 ± 1.4	5.1 ± 0.2	3.6 ± 0.1	17.7 ± 2.2	0.2 ± 0.0	85.1 ± 21.9
G2 500	150.3 ± 1.3 *	6.4 ± 0.7 *	114.4 ± 1.7	5.0 ± 0.2	3.6 ± 0.2	18.0 ± 3.4	0.2 ± 0.0	76.8 ± 21.5
G3 1000	151.6 ± 1.3 ***	6.5 ± 1.3 *	115.3 ± 3.0	5.0 ± 0.2	3.6 ± 0.2	19.2 ± 2.1	0.2 ± 0.0	74.5 ± 17.6
G4 2000	153.1 ± 1.5 ***	6.3 ± 0.5 ***	116.2 ± 1.6	5.1 ± 0.2	3.5 ± 0.1	18.4 ± 1.7	0.2 ± 0.0	91.2 ± 21.0
**Group/** **Dose (mg/kg/Day)**	**Total Bilirubin (mg/dL)**	**Calcium (mg/dL)**	**Phosphate (mg/dL)**	**Total Cholesterol (mg/dL)**	**Triglyceride (mg/dL)**	**Aspartate Aminotransferase (U/L)**	**Alanine Aminotransferase (U/L)**	**Alkaline Phosphatase (U/L)**
G1 0	0.0 ± 0.0	9.1 ± 0.3	7.4 ± 1.0	113.0 ± 19.0	97.9 ± 27.5	88.0 ± 42.6	22.8 ± 5.0	291.1 ± 84.1
G2 500	0.0 ± 0.0	9.1 ± 0.3	7.6 ± 0.8	100.8 ± 15.7	101.7 ± 38.3	91.4 ± 37.6	22.6 ± 4.3	604.4 ± 78.3
G3 1000	0.0 ± 0.0	9.2 ± 0.4	7.5 ± 1.0	106.5 ± 21.1	70.5 ± 22.6 *	106.8 ± 43.1	24.5 ± 4.5	295.7 ± 78.2
G4 2000	0.0 ± 0.0	9.1 ± 0.2	7.8 ± 0.8	120.7 ± 21.7	108.5 ± 30.9	93.5 ± 39.8	24.5 ± 5.9	329.7 ± 99.8
**c. Approximately 5 μm, Male, Hematology Parameter** **s**								
**Group/** **Dose (mg/kg/Day)**	**White blood cell (×10^3^ cells/uL)**	**Red blood cell (×10^6^ cells/uL)**	**Hemoglobin** **(g/dL)**	**Hematocrit** **(%)**	**Mean Corpuscular Volume** **(fL)**	**Mean Corpuscular Hemoglobin** **(pg)**	**Mean Corpuscular Hemoglobin Concentration** **(g/dL)**	**Red Cell Distribution Width** **(%)**
G1 0	3.52 ± 1.58	9.23 ± 1.27	14.5 ± 1.7	46.6 ± 6.0	50.5 ± 0.4	15.7 ± 0.3	31.1 ± 0.6	12.5 ± 0.3
G2 500	3.41 ± 0.65	9.25 ± 0.23	13.8 ± 0.1	45.0 ± 0.3	48.7 ± 1.5	15.0 ± 0.3	30.7 ± 0.4	12.3 ± 0.5
G3 1000	4.25 ± 1.23	9.03 ± 0.17	14.0 ± 0.4	45.4 ± 1.0	50.2 ± 0.3	15.5 ± 0.2	30.8 ± 0.4	13.5 ± 0.4
G4 2000	4.90 ± 0.98	8.74 ± 0.17	13.4 ± 0.4	43.1 ± 1.0	49.6 ± 1.0	15.4 ± 0.5	31.0 ± 0.6	12.5 ± 0.4
**Group/** **Dose (mg/kg/Day)**	**Hemoglobin Distribution Width** **(g/dL)**	**Platelet (×10^3^ cells/uL)**	**Mean Platelet Volume** **(fL)**	**Neutrophil** **(%)**	**Lymphocyte** **(%)**	**Monocyte** **(%)**	**Eosinophil** **(%)**	**Basophil** **(%)**
G1 0	2.35 ± 0.14	844 ± 62	5.3 ± 0.2	26.5 ± 1.7	39.5 ± 9.3	3.4 ± 0.9	30.2 ± 7.0	0.2 ± 0.1
G2 500	2.32 ± 0.06 *	1042 ± 60	4.9 ± 0.1	21.7 ± 6.6	60.2 ± 10.3	2.9 ± 1.9	14.9 ± 16.3	0.1 ± 0.1
G3 1000	2.44 ± 0.21	1150 ± 229	4.7 ± 0.3	28.7 ± 3.3	60.9 ± 3.4	3.1 ± 1.0	6.8 ± 3.1	0.1 ± 0.0
G4 2000	2.34 ± 0.12	1146 ± 91	4.8 ± 0.4	18.9 ± 4.4	67.0 ± 3.9	1.7 ± 0.5	11.8 ± 8.5	0.1 ± 0.0
**Group/** **Dose (mg/kg/Day)**	**Neutrophil** **(×10^3^ cells/uL)**	**Lymphocyte** **(×10^3^ cells/uL)**	**Monocyte (×10^3^ cells/uL)**	**Eosinophil (×10^3^ cells/uL)**	**Basophil (×10^3^ cells/uL)**	**Reticulocyte (×10^9^ cells/L)**	**Reticulocyte (%)**	
G1 0	0.92 ± 0.40	1.46 ± 0.84	0.11 ± 0.04	1.00 ± 0.39	0.01 ± 0.01	427.3 ± 75.7	4.61 ± 0.18	
G2 500	0.91 ± 0.09	2.58 ± 0.43	0.13 ± 0.07	0.81 ± 1.06	0.00 ± 0.01	312.2 ± 56.5 *	3.37 ± 0.58 *	
G3 1000	1.35 ± 0.29	2.93 ± 0.90	0.15 ± 0.05	0.35 ± 0.25	0.01 ± 0.01	388.4 ± 53.7	4.30 ± 0.52	
G4 2000	1.02 ± 0.25	3.60 ± 0.38	0.10 ± 0.04	0.63 ± 0.43	0.01 ± 0.00	332.6 ± 46.2	3.81 ± 0.61	
**d. Approximately 5 μm, Female, Hematology Parameter** **s**								
**Group/** **Dose (mg/kg/Day)**	**White Blood Cell (×10^3^ cells/uL)**	**Red Blood Cell (×10^6^ cells/uL)**	**Hemoglobin** **(g/dL)**	**Hematocrit** **(%)**	**Mean Corpuscular Volume** **(fL)**	**Mean Corpuscular Hemoglobin** **(pg)**	**Mean Corpuscular Hemoglobin Concentration** **(g/dL)**	**Red Cell Distribution Width** **(%)**
G1 0	5.31 ± 2.22	9.47 ± 0.27	15.1 ± 0.3	47.5 ± 1.5	50.1 ± 0.5	15.9 ± 0.2	31.7 ± 0.7	13.3 ± 0.7
G2 500	3.75 ± 0.97	9.26 ± 0.22	14.5 ± 0.3	46.3 ± 0.7	50.0 ± 0.9	15.7 ± 0.5	31.4 ± 0.6	12.8 ± 0.8
G3 1000	7.42 ± 3.21	9.17 ± 0.23	14.5 ± 0.7	45.5 ± 1.9	49.7 ± 1.0	15.8 ± 0.3	31.8 ± 0.5	13.2 ± 0.4
G4 2000	5.18 ± 2.70	9.53 ± 0.35	14.9 ± 0.4	46.9 ± 1.4	49.2 ± 1.1	15.7 ± 0.7	31.8 ± 0.8	13.1 ± 0.2
**Group/** **Dose (mg/kg/Day)**	**Hemoglobin Distribution Width** **(g/dL)**	**Platelet (×10^3^ Cells/uL)**	**Mean Platelet Volume** **(fL)**	**Neutrophil** **(%)**	**Lymphocyte** **(%)**	**Monocyte** **(%)**	**Eosinophil** **(%)**	**Basophil** **(%)**
G1 0	2.41 ± 0.12	872 ± 186	5.1 ± 0.3	15.6 ± 3.5	71.2 ± 8.6	1.9 ± 0.6	10.9 ± 6.5	0.1 ± 0.1
G2 500	2.37 ± 0.10	968 ± 35	5.0 ± 0.2	14.9 ± 2.5	73.7 ± 3.4	1.6 ± 0.3	9.2 ± 5.2	0.1 ± 0.1 *
G3 1000	2.55 ± 0.13	903 ± 94	5.3 ± 0.6	21.6 ± 3.1	64.1 ± 10.0 *	2.2 ± 0.5	11.4 ± 8.1	0.1 ± 0.1
G4 2000	2.49 ± 0.08	849 ± 102	5.7 ± 0.2	18.0 ± 2.7 **	64.0 ± 9.9	1.1 ± 0.5	16.3 ± 8.2	0.3 ± 0.1
**Group/** **Dose (mg/kg/Day)**	**Neutrophil** **(×10^3^ Cells/uL)**	**Lymphocyte** **(×10^3^ cells/uL)**	**Monocyte (×10^3^ Cells/uL)**	**Eosinophil (×10^3^ Cells/uL)**	**Basophil (×10^3^ Cells/uL)**	**Reticulocyte (×10^9^ Cells/L)**	**Reticulocyte (%)**	
G1 0	0.96 ± 0.21	4.72 ± 2.31	0.13 ± 0.07	0.71 ± 0.45	0.01 ± 0.01	371.2 ± 82.3	3.91 ± 0.77	
G2 500	0.64 ± 0.16	3.23 ± 0.95	0.07 ± 0.02	0.40 ± 0.20	0.01 ± 0.01	424.0 ± 20.6	4.58 ± 0.26	
G3 1000	1.75 ± 0.25	5.42 ± 2.20	0.19 ± 0.10	0.88 ± 0.51	0.01 ± 0.01	350.8 ± 21.7	3.83 ± 0.27	
G4 2000	1.13 ± 0.57	4.13 ± 2.25	0.07 ± 0.06	0.93 ± 0.58	0.02 ± 0.01	316.7 ± 26.2	3.32 ± 0.19	
**e. 10–50 μm, Male, Serum Biochemistry Parameters**								
**Group/** **Dose (mg/kg/Day)**	**Sodium (mmol/L)**	**Potassium (mmol/L)**	**Chloride (mmol/L)**	**Total Protein (g/dL)**	**Albumin (g/dL)**	**Blood Urea Nitrogen** **(mg/dL)**	**Creatinine (mg/dL)**	**Glucose (mg/dL)**
G1 0	148.0 ± 1.1	7.8 ± 1.0	111.2 ± 2.1	5.1 ± 0.3	3.2 ± 0.2	23.2 ± 2.9	0.2 ± 0.0	119.4 ± 41.0
G2 500	148.4 ± 1.2	7.3 ± 1.8	112.3 ± 2.1	5.3 ± 0.2	3.4 ± 0.2	21.8 ± 2.4	0.2 ± 0.0	178.9 ± 36.8
G3 1000	149.2 ± 1.1	7.8 ± 1.2	113.5 ± 1.3	5.1 ± 0.2	3.1 ± 0.2	19.7 ± 3.7	0.2 ± 0.0	159.8 ± 49.5
G4 2000	150.7 ± 1.6	7.3 ± 0.9	112.9 ± 1.8	5.2 ± 0.2	3.3 ± 0.2	26.1 ± 5.7	0.2 ± 0.0	123.7 ± 36.5
**Group/** **Dose (mg/kg/Day)**	**Total Bilirubin (mg/dL)**	**Calcium (mg/dL)**	**Phosphate (mg/dL)**	**Total Cholesterol (mg/dL)**	**Triglyceride (mg/dL)**	**Aspartate Aminotransferase (U/L)**	**Alanine Aminotransferase (U/L)**	**Alkaline Phosphatase (U/L)**
G1 0	0.1 ± 0.0	8.5 ± 0.3	8.5 ± 1.1	158.8 ± 24.2	117.5 ± 26.9	81.9 ± 46.1	26.6 ± 8.9	188.5 ± 63.4
G2 500	0.1 ± 0.0	8.6 ± 0.3	7.1 ± 1.5	151.5 ± 18.7	125.5 ± 51.5	64.4 ± 19.6	28.0 ± 12.5	235.6 ± 86.4
G3 1000	0.1 ± 0.0	8.5 ± 0.2	7.5 ± 1.3	146.4 ± 23.0	127.2 ± 78.5	63.3 ± 31.5	21.3 ± 5.4	152.1 ± 53.1
G4 2000	0.1 ± 0.1	8.6 ± 0.3	7.8 ± 1.2	155.0 ± 19.8	108.5 ± 26.5	78.2 ± 24.9	39.3 ± 30.8	200.2 ± 72.5
**f. 10–** **50 μm, Female, Serum Biochemistry Parameters**								
**Group/** **Dose (mg/kg/Day)**	**Sodium (mmol/L)**	**Potassium (mmol/L)**	**Chloride (mmol/L)**	**Total Protein (g/dL)**	**Albumin (g/dL)**	**Blood Urea Nitrogen** **(mg/dL)**	**Creatinine (mg/dL)**	**Glucose (mg/dL)**
G1 0	151.8 ± 3.4	6.3 ± 0.9	114.1 ± 2.9	5.0 ± 0.3	3.6 ± 0.2	18.7 ± 1.9	0.2 ± 0.0	115.6 ± 34.8
G2 500	155.9 ± 3.7 *	6.5 ± 0.6	117.9 ± 4.5 *	5.0 ± 0.3	3.5 ± 0.2	16.1 ± 4.3	0.2 ± 0.0	147.4 ± 57.4
G3 1000	159.1 ± 5.0 **	7.2 ± 1.9	120.5 ± 3.3 ***	5.1 ± 0.3	3.7 ± 0.2	19.5 ± 3.3	0.2 ± 0.0	141.0 ± 48.4
G4 2000	152.2 ± 2.7	7.5 ± 2.0	117.1 ± 2.3 *	5.2 ± 0.4	3.6 ± 0.2	14.7 ± 3.2 **	0.2 ± 0.0	172.8 ± 30.5 **
**Group/** **Dose (mg/kg/Day)**	**Total Bilirubin (mg/dL)**	**Calcium (mg/dL)**	**Phosphate (mg/dL)**	**Total Cholesterol (mg/dL)**	**Triglyceride** **(mg/dL)**	**Aspartate Aminotransferase (U/L)**	**Alanine Aminotransferase** **(U/L)**	**Alkaline Phosphatase (U/L)**
G1 0	0.0 ± 0.0	9.4 ± 0.4	7.9 ± 1.2	105.4 ± 25.6	50.2 ± 14.6	82.1 ± 79.1	45.6 ± 69.4	301.6 ± 111.1
G2 500	0.0 ± 0.0	9.7 ± 0.3	7.9 ± 0.8	113.8 ± 25.6	55.8 ± 19.3	53.8 ± 6.3	23.4 ± 12.8	262.4 ± 35.0
G3 1000	0.0 ± 0.0	10.0 ± 0.5 *	8.1 ± 1.4	114.6 ± 18.1	62.4 ± 27.0	54.2 ± 7.8	22.4 ± 8.6	333.0 ± 88.1
G4 2000	0.0 ± 0.0	9.5 ± 0.4	8.4 ± 1.1	95.6 ± 25.3	34.4 ± 20.4	113.6 ± 115.1	24.6 ± 12.3	261.4 ± 55.2
**g. 10–50 μm, Male, Hematology Parameters**								
**Group/** **Dose (mg/kg/Day)**	**White Blood Cell (×10^3^ cells/uL)**	**Red Blood Cell (×10^6^ cells/uL)**	**Hemoglobin** **(g/dL)**	**Hematocrit** **(%)**	**Mean Corpuscular Volume** **(fL)**	**Mean Corpuscular Hemoglobin** **(pg)**	**Mean Corpuscular Hemoglobin Concentration** **(g/dL)**	**Red Cell Distribution Width** **(%)**
G1 0	5.50 ± 1.88	9.25 ± 0.58	14.4 ± 0.9	47.0 ± 3.2	50.8 ± 1.33	15.6 ± 0.3	30.7 ± 0.2	12.9 ± 0.7
G2 500	5.75 ± 0.94	8.93 ± 0.09	13..8 ± 0.3	44.4 ± 1.0	49.7 ± 1.4	15.4 ± 0.4	31.0 ± 0.7	12.9 ± 0.2
G3 1000	6.30 ± 2.32	8.90 ± 0.49	13.6 ± 0.4 *	44.0 ± 1.5	49.4 ± 1.1	15.3 ± 0.4	31.0 ± 0.4	12.9 ± 0.6
G4 2000	4.68 ± 0.59	8.36 ± 1.10	13.3 ± 1.1	42.3 ± 3.9	50.8 ± 2.0	16.0 ± 0.9	31.4 ± 0.6	12.8 ± 0.1
**Group/** **Dose (mg/kg/Day)**	**Hemoglobin Distribution Width** **(g/dL)**	**Platelet (×10^3^ Cells/uL)**	**Mean Platelet Volume** **(fL)**	**Neutrophil** **(%)**	**Lymphocyte** **(%)**	**Monocyte** **(%)**	**Eosinophil** **(%)**	**Basophil** **(%)**
G1 0	2.47 ± 0.21	1047 ± 86	5.1 ± 0.3	17.4 ± 3.1	66.6 ± 4.7	3.4 ± 0.3	12.0 ± 5.6	0.2 ± 0.2
G2 500	2.34 ± 0.12	983 ± 54	4.9 ± 0.7	21.2 ± 6.6	65.3 ± 6.6	3.6 ± 1.2	9.4 ± 8.9	0.2 ± 0.2
G3 1000	2.43 ± 0.06	1080 ± 232	4.9 ± 0.3	17.1 ± 4.3	70.4 ± 3.4	2.2 ± 1.0	9.8 ± 2.5	0.1 ± 0.0
G4 2000	2.39 ± 0.15	1101 ± 167	4.9 ± 0.2	40.8 ± 36.4	41.5 ± 35.4	2.5 ± 1.0	14.9 ± 9.3	0.1 ± 0.0
**Group/** **Dose (mg/kg/Day)**	**Neutrophil** **(×10^3^ Cells/uL)**	**Lymphocyte** **(×10^3^ Cells/uL)**	**Monocyte (×10^3^ Cells/uL)**	**Eosinophil (×10^3^ Cells/uL)**	**Basophil (×10^3^ Cells/uL)**	**Reticulocyte (×10^9^ Cells/L)**	**Reticulocyte (%)**	
G1 0	4.33 ± 1.69	0.22 ± 0.06	0.69 ± 0.16	0.03 ± 0.01	0.01 ± 0.01	364.7 ± 78.9	3.92 ± 0.62	
G2 500	1.48 ± 0.35	4.65 ± 1.04	0.26 ± 0.11	0.66 ± 0.59	0.02 ± 0.01	326.9 ± 59.5	3.66 ± 0.70	
G3 1000	1.26 ± 0.31	5.52 ± 2.35	0.16 ± 0.07	0.81 ± 0.51	0.01 ± 0.00	333.6 ± 18.7	3.75 ± 0.28	
G4 2000	1.85 ± 1.55	2.06 ± 1.90	0.12 ± 0.07	0.67 ± 0.36	0.00 ± 0.01	355.9 ± 26.9	4.33 ± 0.86	
**h. 10–** **50** **μm, Female, Hematology Parameters**								
**Group/** **Dose (mg/kg/Day)**	**White Blood Cell (×10^3^ Cells/uL)**	**Red Blood Cell (×10^6^ cells/uL)**	**Hemoglobin** **(g/dL)**	**Hematocrit** **(%)**	**Mean Corpuscular Volume** **(fL)**	**Mean Corpuscular Hemoglobin** **(pg)**	**Mean Corpuscular Hemoglobin Concentration** **(g/dL)**	**Red Cell Distribution Width** **(%)**
G1 0	4.95 ± 2.22	9.50 ± 0.66	15.1 ± 0.2	47.5 ± 0.7	50.1 ± 2.7	15.9 ± 0.9	31.8 ± 0.1	12.8 ± 1.0
G2 500	7.77 ± 2.80	9.50 ± 0.90	14.8 ± 1.1 *	47.2 ± 3.3 *	49.8 ± 2.2	15.5 ± 0.5	31.2 ± 0.3	12.7 ± 0.6
G3 1000	5.61 ± 0.64	9.04 ± 0.60	14.2 ± 0.9	45.6 ± 3.0	50.4 ± 0.9	15.7 ± 0.1	31.1 ± 0.5	13.6 ± 2.4
G4 2000	3.49 ± 1.89	9.91 ± 0.60	15.6 ± 0.7	49.8 ± 2.6	50.2 ± 1.0	15.7 ± 0.3	31.4 ± 0.5	12.5 ± 0.2
**Group/** **Dose (mg/kg/Day)**	**Hemoglobin Distribution Width** **(g/dL)**	**Platelet (×10^3^ Cells/uL)**	**Mean Platelet Volume** **(fL)**	**Neutrophil** **(%)**	**Lymphocyte** **(%)**	**Monocyte** **(%)**	**Eosinophil** **(%)**	**Basophil** **(%)**
G1 0	2.34 ± 0.02	915 ± 12	5.0 ± 0.2	15.2 ± 3.2	67.5 ± 9.6	1.8 ± 0.4	15.1 ± 8.4	0.1 ± 0.0
G2 500	2.29 ± 0.11	942 ± 78	5.1 ± 0.3	18.3 ± 4.0	68.9 ± 3.1	1.5 ± 0.9	10.7 ± 6.4	0.2 ± 0.1
G3 1000	2.45 ± 0.38	977 ± 193	4.9 ± 0.5	16.3 ± 2.8	74.5 ± 2.0	1.4 ± 0.3	7.1 ± 3.4	0.1 ± 0.0
G4 2000	2.29 ± 0.10	939 ± 212	5.2 ± 0.5	17.4 ± 3.2	69.2 ± 13.0	1.3 ± 0.4	11.5 ± 10.3	0.1 ± 0.1
**Group/** **Dose (mg/kg/Day)**	**Neutrophil** **(×10^3^ Cells/uL)**	**Lymphocyte** **(×10^3^ Cells/uL)**	**Monocyte (×10^3^ Cells/uL)**	**Eosinophil (×10^3^ Cells/uL)**	**Basophil (×10^3^ Cells/uL)**	**Reticulocyte (×10^9^ Cells/L)**	**Reticulocyte (%)**	
G1 0	0.80 ± 0.39	3.75 ± 2.27	0.09 ± 0.04	0.65 ± 0.10	0.00 ± 0.01	396.3 ± 132.9	4.21 ± 1.58	
G2 500	1.57 ± 0.74	5.75 ± 1.99	0.12 ± 0.04	0.78 ± 0.22	0.01 ± 0.01	425.9 ± 153.7	4.49 ± 1.48	
G3 1000	1.05 ± 0.29	4.78 ± 0.81	0.09 ± 0.01	0.44 ± 0.16	0.01 ± 0.01	508.2 ± 397.6	5.82 ± 4.89	
G4 2000	0.79 ± 0.42	3.08 ± 1.82	0.06 ± 0.04	0.54 ± 0.50	0.01 ± 0.01	325.0 ± 34.2	3.29 ± 0.42	

## Data Availability

The data that support the findings of this study are available from the corresponding author upon reasonable request.

## Supplementary Materials

The following supporting information can be downloaded at: https://www.mdpi.com/article/10.3390/polym14112220/s1; Table S1: Male and female organ weight in the 28-day repeated-oral-dose toxicity study.
