# Supplementary material for: In Vivo Toxicity and Pharmacokinetics of Polytetrafluoroethylene Microplastics in ICR Mice
Source: Polymers (Basel). 2022 May 30;14(11):2220. doi: 10.3390/polym14112220 (PMC9182653; doi:10.3390/polym14112220)
Supplement: Supplementary file 1 [file polymers-14-02220-s001.zip › polymers-1654780-supplementary.pdf]

Table S1. Male and female organ weights in the 28-day repeated oral dose toxicity study of approximately 5 μm (a, b) and 10–50 μm (c, d). The results are expressed as the mean ± SD. *$p < 0.05$, **$p < 0.01$, ***$p < 0.001$ vs. 0.

a. approximately 5 μm, male, organ weights

Group / Dose (mg/kg/day)	Brain	Heart	Liver	Kidney	Spleen	Testis	Epididymis
G1 0	0.47±0.03	0.18±0.03	1.43±0.15	0.44±0.05	0.09±0.02	0.25±0.05	0.09±0.01
G2 500	0.47±0.02	0.18±0.03	1.36±0.09	0.45±0.03	0.09±0.01	0.27±0.04	0.09±0.02
G3 1,000	0.47±0.03	0.18±0.03	1.37±0.09	0.44±0.05	0.10±0.02	0.25±0.04	0.09±0.01
G4 2,000	0.48±0.03	0.18±0.02	1.33±0.11	0.46±0.04	0.09±0.02	0.26±0.04	0.10±0.03

b. approximately 5 μm, female, organ weights

Group / Dose (mg/kg/day)	Brain	Heart	Liver	Kidney	Spleen	Testis
G1 0	0.47±0.03	0.15±0.02	1.12±0.16	0.30±0.03	0.10±0.02	0.017±0.003
G2 500	0.46±0.03	0.14±0.01	1.02±0.09	0.28±0.02	0.09±0.02	0.018±0.005
G3 1,000	0.48±0.03	0.14±0.01	1.02±0.14	0.28±0.03	0.09±0.01	0.017±0.007
G4 2,000	0.45±0.02	0.17±0.12	1.01±0.12	0.29±0.02	0.10±0.02	0.015±0.005

c. 10-50 μm, male, organ weights

Group / Dose (mg/kg/day)	Brain	Heart	Liver	Kidney	Spleen	Testis	Epididymis
G1 0	0.46±0.02	0.19±0.03	1.35±0.14	0.44±0.07	0.10±0.02	0.25±0.03	0.09±0.02
G2 500	0.46±0.04	0.16±0.02*	1.48±0.25	0.42±0.04	0.08±0.01*	0.23±0.03	0.08±0.02
G3 1,000	0.47±0.02	0.18±0.02	1.46±0.07*	0.42±0.04	0.09±0.02	0.25±0.02	0.09±0.01
G4 2,000	0.48±0.04	0.17±0.02	1.38±0.11	0.44±0.03	0.10±0.02	0.25±0.04	0.08±0.01

d. 10-50 μm, male, organ weights

Group / Dose (mg/kg/day)	Brain	Heart	Liver	Kidney	Spleen	Ovary
G1 0	0.48±0.02	0.15±0.02	1.14±0.11	0.29±0.03	0.09±0.02	0.017±0.004
G2 500	0.46±0.02	0.13±0.02*	1.07±0.11	0.28±0.02	0.11±0.04	0.015±0.004
G3 1,000	0.48±0.03	0.14±0.02	1.05±0.12	0.29±0.03	0.09±0.03	0.015±0.003
G4 2,000	0.47±0.02	0.14±0.02	1.04±0.05*	0.27±0.03	0.09±0.02	0.016±0.006
